# Coronary Artery Calcium Scans by Area Deprivation Index in a Multicenter Health System

**DOI:** 10.1016/j.jacadv.2026.102657

**Published:** 2026-03-16

**Authors:** Grant Stoltman, Gretchen Benson, Lillian Cichon, Larissa Stanberry, Michael D. Miedema, Retu Saxena

**Affiliations:** a Minneapolis Heart Institute Foundation, Minneapolis, Minnesota, USA; b Allina Health Minneapolis Heart Institute, Minneapolis, Minnesota, USA

**Keywords:** coronary artery calcium, preventive cardiology, social determinants of health, socioeconomic disparities

Cardiovascular disease (CVD) remains a leading cause of morbidity and mortality worldwide.^1^ Coronary artery calcium (CAC) is a well-established, noninvasive clinical tool for cardiovascular risk assessment and a strong, independent predictor of future atherosclerotic CVD events.^2^ Incorporation of CAC scoring into clinical decision-making can improve risk stratification beyond traditional risk factors. Although CAC-guided risk assessment can influence preventive therapies, its use depends on access and utilization within health systems.

**What is the clinical question being addressed?**

Is neighborhood socioeconomic deprivation, measured by ADI, associated with the use of coronary artery calcium scanning?

**What is the main finding?**

Patients with the greatest socioeconomic disadvantage had the highest coronary artery calcium scores but the lowest coronary artery calcium testing representation.

Social and environmental conditions contribute meaningfully to cardiovascular risk and related inequities.^3^ Socioeconomic disadvantage, in particular, has been consistently associated with a greater burden of CVD.^4^ The Area Deprivation Index (ADI) is a validated, neighborhood-level measure of socioeconomic disadvantage that incorporates income, education, employment, and housing quality, and has been associated with adverse health outcomes, higher mortality, and disparities in access to care.^5^

Although higher ADI has been linked to worse health outcomes, its relationship with the utilization of cardiovascular preventive imaging remains poorly understood. Understanding whether socioeconomic factors are associated with differential CAC use is important as unequal utilization of this risk stratification tool could contribute to downstream disparities in CVD prevention and outcomes. The purpose of this study was to evaluate the relationship between an individual’s socioeconomic disadvantage and the utilization of CAC scans to identify potential disparities in the use of this preventive tool.

## Methods

This retrospective cohort study consisted of 5,900 individuals who underwent noncontrast computed tomography for CAC scoring between April 2024 and April 2025. Scans were a part of a physician- or self-referred cardiovascular risk assessment, each with a $100 out-of-pocket cost. Patient data were collected through electronic health records from 8 outpatient imaging sites across a large Midwestern Healthcare system. The Allina Health Institutional Review Board determined this project exempt from Institutional Review Board review.

Patient residential addresses and scanner locations were mapped to ADI values corresponding to state deciles, with 1 representing low socioeconomic disadvantage and 10 representing highly disadvantaged. Deciles were grouped into tertiles representing low (1-3), mid (4-6), and high (7-10) socioeconomic disadvantage.

Patients with missing residential address data or incomplete CAC results were excluded from the analysis. CAC prevalence was defined as CAC >0. Distributions of patients across ADI tertiles were compared using the Pearson chi-square test. Associations between ADI tertile and CAC presence were evaluated using univariable logistic regression and a series of multivariable logistic regression models incorporating site of scan, demographic variables (age, sex, race/ethnicity, body mass index), and clinical cardiovascular risk factors (diabetes, hypertension, hyperlipidemia, family history of CVD, and smoking status), with results reported as ORs and 95% CIs. Statistical significance was defined as *P* < 0.05. All statistical analyses were completed using R v 4.4.1 (R Core Team) in RStudio 2025.05.1 environment (Posit Software, PBC).

## Results

A total of 5,748 patients were included in the analysis during the study period. The cohort median age was 60 years (Q1-Q3: 52-66 years), and 47.4% of patients were males. Racial and ethnic composition was predominantly Caucasian (94.3%), with smaller proportions of Asian/Pacific Islander (3.4%), African American (1.4%), and Hispanic individuals (0.9%). The median patient ADI was 5 (Q1-Q3: 2-7), and scanners were located in neighborhoods with ADI deciles ranging from 3 to 9.

**Figure 1:**
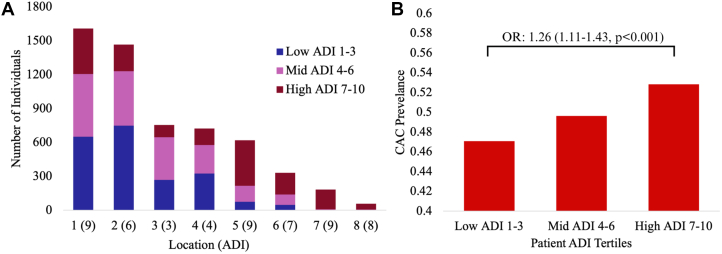
**Population Distribution and Coronary Artery Calcium Prevalence by Patient ADI Tertiles** (A) Across 8 scanning sites, the proportions of patients in low-, mid-, and high-ADI tertiles varied significantly (chi-square = 1,344.2; *P* < 0.001). (B) Prevalence of CAC>0 increased across tertiles, with patients in the high-ADI tertile having greater odds compared with those in the low-ADI tertile (OR: 1.26; 95% CI: 1.11-1.43; *P* < 0.001). ADI = Area Deprivation Index.

Overall, 49.7% of individuals exhibited prevalent CAC. The median CAC was greatest in the high tertile at 2 (Q1-Q3: 0-91), compared with 0 (Q1-Q3: 0-43.5) and 0 (Q1-Q3: 0-53) in the low- and mid-tertiles, respectively. In univariable analysis, patients in the high-ADI tertile were more likely to have prevalent CAC compared with those in the low-ADI tertile (OR: 1.26; 95% CI: 1.11-1.43; *P* < 0.001) (Figure 1). The unadjusted association attenuated after adjustment for demographics and clinical risk factors and was no longer statistically significant in the fully adjusted model (OR: 1.11; 95% CI: 0.79-1.56; *P* = 0.53).

CAC scan distribution varied significantly across ADI tertiles, with 37.0% of patients in the low tertile, 33.4% in the mid tertile, and 29.6% in the high tertile (chi-square = 1,344.2; *P* < 0.001). Compared to patients with low ADIs, those from high-ADI neighborhoods were under-represented among individuals who underwent CAC scanning.

## Discussion

In this multisite health system analysis of CAC scanning patterns, patients from socioeconomically disadvantaged neighborhoods had higher odds of atherosclerosis, largely mediated by patient-specific factors. Despite these increased odds, patients with high ADI accounted for a disproportionately smaller share of those undergoing CAC scans.

Disparities in CAC screening in socioeconomically disadvantaged populations may limit opportunities for early detection of subclinical atherosclerosis as well as the timely initiation of evidence-based preventive strategies and therapies. Without timely risk identification, atherosclerosis may progress undetected and contribute to greater cardiovascular risk over time. Previous studies have shown that removing barriers to CAC testing, such as out-of-pocket costs, is associated with increased recruitment of individuals from socially vulnerable subgroups.^4^ Therefore, promoting equitable access to CAC scanning may represent an important strategy for population-based CVD prevention initiatives.

### Study strengths and limitations

This study is strengthened by its large, multisite cohort and use of a validated neighborhood-level measure of socioeconomic disadvantage. Limitations include the retrospective, observational design, necessitating future mechanistic studies with expanded multivariable adjustment to better understand the pathways underlying the association between socioeconomic deprivation and CAC scanning.

## Conclusions

In a large, multisite health system, patients from more socioeconomically disadvantaged neighborhoods had higher odds of CAC but were less likely to undergo CAC scanning. These findings highlight a potential disparity in the utilization of a cardiovascular risk tool, and addressing this gap may be important for promoting equitable risk assessment and prevention efforts.

## Funding Support and Author Disclosures

The partnership of Penny and Lee Anderson and the staff at the Minneapolis Heart Institute Foundation’s Penny Anderson Women’s Cardiovascular Center helped support this research project. The authors have reported that they have no relationships relevant to the contents of this paper to disclose.
